# A Method for Compensating Hemoglobin Interference in Total Serum Bilirubin Measurement Using a Simple Two-Wavelength Reflectance Photometer

**DOI:** 10.3390/s24206749

**Published:** 2024-10-20

**Authors:** Lorenzo Zucchini, Carlos Daniel Coda Zabetta, Miloš Ajčević, Agostino Accardo

**Affiliations:** 1 Department of Engineering and Architecture, University of Trieste, 34127 Trieste, Italy; majcevic@units.it (M.A.); accardo@units.it (A.A.); 2 Bilimetrix s.r.l., 34149 Trieste, Italy

**Keywords:** bilirubin, hemoglobin, neonatal hyperbilirubinemia, LMICs

## Abstract

Neonatal hyperbilirubinemia (NH) is a common condition in newborns, with elevated bilirubin levels potentially causing neurological damage or death. Accurate and timely measurements of total serum bilirubin are essential to prevent these outcomes. Direct spectrophotometry, a reliable method for measuring bilirubin, is particularly useful in constrained settings due to its potential for portable low-cost instrumentation. However, this method is susceptible to interference from hemoglobin, often present due to hemolysis. Typically, this interference is reduced using complex optical filters, reagents, multiple wavelengths, or combinations thereof, which increase costs and complexity while reducing usability. This study presents a hemoglobin compensation algorithm applied to a simple, portable, two-wavelength (465 and 590 nm) reflectance photometer designed to receive 30 µL of plasma or whole blood samples and perform the measurement without any reagents. Testing across five bilirubin and hemoglobin levels (4.96 to 28 mg/dL and 0.06 to 0.99 g/dL, respectively) demonstrated that the algorithm effectively reduces hemoglobin interference and overestimation errors. The overall root mean square error was reduced from 4.86 to 1.45 mg/dL, while the measurement bias decreased from −4.46 to −0.10 mg/dL. This substantial reduction in overestimation errors supports future clinical trials with neonatal blood samples.

## 1. Introduction

Bilirubin is the final breakdown product of erythrocyte metabolism, released into plasma as unconjugated bilirubin (UCB), which binds to albumin and is excreted in bile [1,2]. Hyperbilirubinemia, marked by elevated UCB, often results from hemolysis or impaired conjugation with albumin. When hyperbilirubinemia occurs in a neonatal patient, it is referred to as neonatal hyperbilirubinemia (NH). While 60–80% of newborns experience temporary physiological NH within the first days of life, usually without needing treatment [3,4,5], about one in ten newborns develop pathological NH, which poses a risk of severe neurological damage, kernicterus, or death [2,3,4,5]. Low- to middle-income countries (LMICs) bear the greatest burden of NH [6,7,8,9,10,11], with most cases of permanent neurological damage or death from kernicterus occurring in these regions. Despite NH being a well-known issue, reliable data on its incidence and consequences are scarce and difficult to generalize. Two recent meta-analyses addressed the challenge of analyzing available data, revealing that severe NH is 11 times more common in poorer countries compared to wealthier ones. In 2010, stillbirths due to kernicterus were 119 per 100,000 in low-income countries compared to just 1 per 100,000 in high-income countries [7,10]. Both studies agreed that the real burden of NH is likely underestimated, especially in LMICs, where data quality and key clinical or demographic details are often lacking. This disparity reflects a multifaceted issue, including inadequate infrastructure, inefficient technologies, poor maternal education, and limited healthcare accessibility [11,12,13,14,15]. A timely and quantitative bilirubin assessment in the early days of life is crucial [16,17,18]. Consequently, major clinical guidelines for NH diagnosis and treatment emphasize the need for precise and prompt bilirubin screening, with diagnostic evaluations based on total serum bilirubin levels in the blood [5,19,20,21,22,23]. While non-invasive methods like transcutaneous bilirubinometers can estimate bilirubin concentration for screening purposes [24,25], diagnosis and subsequent clinical decisions must rely on quantitative, invasive, and blood-based measurements.

Direct spectrophotometry is a valuable method for measuring total serum bilirubin and is an established technique for both laboratory and point-of-care or field instrumentation [26,27,28,29,30,31]. The recent literature reports various examples of small, portable, economical, and easy-to-use devices that address the specific needs and constraints of LMICs. However, a major limitation of direct spectrophotometry is interference from other pigments in the sample, with hemoglobin being among the most common [32,33,34,35,36]. Hemoglobin may be present in plasma as a result of hemolysis, which can occur in vivo as a result of enzyme deficiencies, infections, or other pathological conditions [37,38]. Alternatively, hemolysis may occur in vitro due to mechanical damage, improper collection techniques, or issues with the equipment used for collection [39,40,41]. This issue is particularly critical in neonatal care. Capillary heel stick blood collection, favored for being less invasive and painful, is widely utilized; however, it is strongly associated with higher levels of hemolysis [35,40,42]. In LMICs, where there is a shortage of skilled professionals and well-defined protocols for sample collection and handling, the likelihood of in vitro hemolysis is further exacerbated.

Laboratory-grade instruments utilizing direct spectrophotometry compensate for interfering analytes by employing reagents or irradiating the sample with multiple wavelengths, up to 512 [30,31,43,44]. While these techniques provide more robust and accurate results, they also introduce greater complexity and increase the size and cost of the instrumentation, as well as the expense of individual measurements. Furthermore, these techniques restrict their application in well-equipped facilities, limiting their accessibility in resource-constrained settings. This contrasts with the recent literature focusing on the spread of diagnostic devices and technologies in limited resource settings [45,46,47]. The World Health Organization’s A.S.S.U.R.E.D. criteria outline fundamental design principles that diagnostic devices should meet to be effective in harsh and under-resourced environments, namely that devices be affordable, sensitive, specific, user-friendly, rapid and robust, equipment-free, and deliverable.

In summary, addressing the challenges of LMICs requires that measurement devices remain simple and economical while maintaining sufficient accuracy for reliable diagnostics with the capability to deliver results quickly and close to the patient. In this work, we present a method for compensating for hemoglobin interference in the measurement of total serum bilirubin using a simple reflectance photometer. This instrument operates with two wavelengths—465 nm and 590 nm—where the former is employed to measure bilirubin concentration, while the latter is used to determine hemoglobin levels. The photometer requires only 30 µL of plasma to perform a measurement, and no reagents are necessary. A panel of plasma samples was prepared to encompass a wide range of bilirubin concentrations from 4.96 to 28.00 mg/dL. Additionally, these bilirubin samples were divided into groups with varying degrees of hemolysis, ranging from 0.06 to 0.99 g/dL. To demonstrate the performance and applicability of the proposed method, results are presented both before and after hemoglobin compensation, and their implications are discussed.

## 2. Materials and Methods

### 2.1. The Measuring Setup

**Figure 1:**
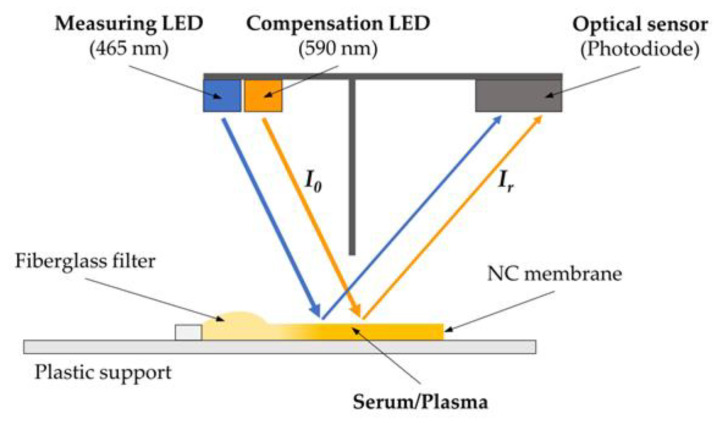
A schema of the reflectance photometer used in the experiments. The two discrete LEDs irradiate the sample after membrane saturation is detected, then the photodiode inside the optical chamber measures the intensity of reflected light.

A two-wavelength reflectance photometer was employed to measure bilirubin and hemoglobin in the samples (Figure 1). The device used two distinct commercial LEDs to illuminate the sample at wavelengths of 465 nm and 590 nm, respectively (KPHHS-1005QBC-D-V and KA-1608SYSK, Kingbright, Taiwan). Reflected light intensity at each wavelength was detected by a photodiode (TEMD6200FX01, Vishay Intertechnology, Inc., Malvern, Pennsylvania, USA). The entire setup, including the sample, was housed within a dark chamber to prevent interference from external light and limit sample degradation. Essential electronic components for signal acquisition and digitalization were also incorporated. The angle of incidence of light on the sample significantly influenced the measurement results and system accuracy. This parameter was carefully considered in selecting the LED lens geometry, with flat transparent lenses chosen for their uniform light spatial distribution at relatively wide angles. Furthermore, the optical chamber’s geometry, including the arrangement and angle of the LED light sources in relation to the membrane surface, was fixed and kept constant throughout all tests. As a result, the influence of the incident angle could be disregarded. The 30 µL plasma samples were applied to disposable lateral flow test strips, which consisted of a fiberglass filter and a nitrocellulose membrane mounted on a plastic support. The fiberglass filter served to block and retain any red blood cell or fragment that might be present in the sample, thereby preventing their entry into the nitrocellulose membrane. As no light passed through the plastic support, the unabsorbed light was reflected in multiple directions within the optical chamber, enabling the photodiode to measure the reflected intensity.

For each test, a new lateral flow test strip was inserted into the optical chamber and its blank reflectance was measured. Then, the plasma sample was deposited on the filter. The system was designed to detect the filling process of the membrane and perform the measurement once complete saturation was perceived. The membrane filling detection process was carried out using the compensation wavelength of 590 nm. This ensured that the measurement wavelength of 465 nm was only used for the actual bilirubin measurement, preventing the sample from being exposed to light near its absorption peak, thereby avoiding degradation. The reflectance intensities at both wavelengths, and thus the result of a single test, were normalized to the blank value to compensate for any differences between test strips. As a result of this normalization process, the reflectance values were expressed as unitless numbers. The photometer performed two consecutive measurements for each test, which were then averaged. At the end of each test, the lateral flow test strip was examined to detect any abnormal conditions or an incomplete saturation of the membrane; if such issues were observed, the test was repeated.

### 2.2. Test Samples

A panel including five different levels of bilirubin concentration and five different levels of hemoglobin concentration was prepared immediately before the experiment.

Commercially purified bilirubin (Bilirubin powder ≥ 98%–Code B4126, Merck KGaA, Darmstadt, Germany) was dissolved in dimethyl sulfoxide to prepare a stock bilirubin solution. Simultaneously, 12 mL of blood was drawn from an adult volunteer into a tube containing K3 EDTA as an anticoagulant. After centrifugation, five plasma aliquots were transferred to separate test tubes. Varying amounts of the bilirubin stock solution were added to each tube to achieve different bilirubin concentrations. To create a stock hemoglobin solution, the same blood sample was lysed and centrifuged again to remove lysed cell fragments, and the resulting hemoglobin-rich plasma was used to prepare five distinct hemoglobin concentrations for each test tube. The hemoglobin concentration in each sample was confirmed using a commercial colorimetric assay (Hemoglobin Colorimetric Method–Code HG1539, Randox Laboratories, Ltd., Crumlin, United Kingdom). The final panel of samples, shown in Table 1, was tested in five replicates per sample.

### 2.3. Hemoglobin Interference Compensation Algorithm

**Figure 2:**
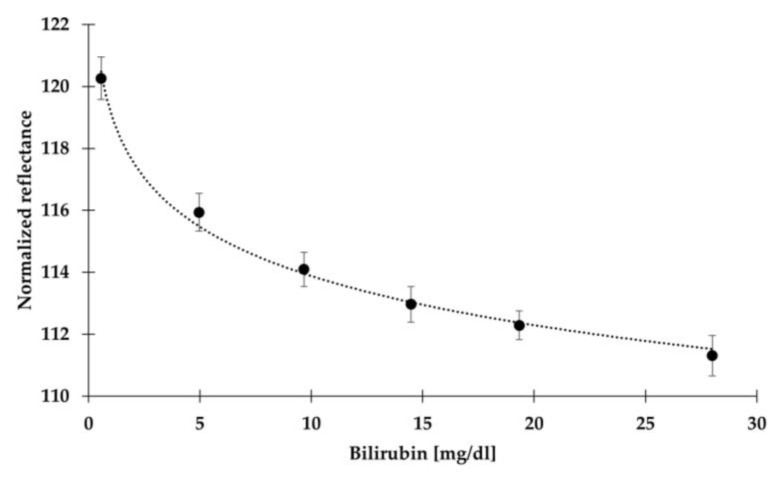
The exponential law used to approximate the relationship between bilirubin and normalized reflectance (dimensionless number). Black dots represent the averaged values for each bilirubin concentration (0.56 to 28.00 mg/dL). Error bars represent the standard deviation for each level. The normalized reflectance values exceed 100 due to the normalization procedure; blank reflectance is measured using the compensation wavelength of 590 nm, which due to the current supply of the LED, has a lower luminous intensity compared to the measurement wavelength of 465 nm. Consequently, for all tested bilirubin levels, the blank reflectance values used for normalization are lower than the actual reflectance values recorded during the measurement at 465 nm, resulting in values above 100.

The reflectance photometer’s response to different bilirubin concentrations was evaluated by testing non-hemolyzed samples across a broad range of bilirubin levels for this experiment. Specifically, the 465 nm wavelength was used due to its proximity to the bilirubin absorption peak [48]. An exponential function was employed to approximate the relationship between the 465 nm reflectance and bilirubin concentration, creating a reflectance–bilirubin calibration curve for estimating bilirubin concentration, as shown in Figure 2. In a previous proof-of-concept study [49], the same reflectance photometer and test strips were used to explore the possibility of achieving compensation using just two wavelengths; however, in such instrument, the compensation wavelength was 575 nm instead of 590 nm. This preliminary effort was helpful in confirming the feasibility of this approach despite its limited accuracy in compensation due to a non-optimal choice of the compensation wavelength. In particular, 570 nm showed a slight sensitivity to bilirubin, leading to the impossibility of identifying a unique compensation law dependent only on hemoglobin concentration. Further preliminary research demonstrated that while at 465 nm, significant absorption is observed for both bilirubin and hemoglobin; at 590 nm, absorption is predominantly attributed to hemoglobin with bilirubin’s effect being theoretically negligible [50]. Consequently, the presence of a specific concentration of hemoglobin leads to a noticeable reduction in reflectance at both wavelengths (indicated as ΔR_590_ and ΔR_465_, respectively) compared to a non-hemolyzed sample, which would cause a measurable decrease in reflectance only at 465 nm. In order to accurately measure bilirubin concentration without interference from hemoglobin at 465 nm, it is essential to establish a relationship between ΔR_590_ and ΔR_465_.

Reflectance values for non-hemolyzed samples, indicated as B1H1, B2H1, …, B5H1 in Table 1, serve as reference points for 590 and 465 nm wavelengths, referred to as R_590, ref_ and R_465, ref_, respectively. For each of the other tests, ΔR_590_ and ΔR_465_ are calculated as reported in Equation (1) and Equation (2), where R_590, [Hb]_ and R_465, [Hb]_ represent the measured reflectance values for a specific hemoglobin concentration at each respective wavelength.

ΔR_590_ = R_590, ref_ − R_590, [Hb]_ (1)

ΔR_465_ = R_465, ref_ − R_465, [Hb]_ (2)

Following this, ΔR_590_ and ΔR_465_ are correlated using a linear function. This method enables the association of a specific difference between R_590_**_, [_**_Hb]_ and R_590, ref_ with the corresponding difference between R_465, [Hb]_ and R_465, ref_, allowing the determination of the reflectance change at 465 nm induced by hemoglobin. Subsequently, ΔR_465_ is added to the measured reflectance value, and the bilirubin concentration is calculated using the established exponential calibration law. This process accounts for hemoglobin interference by leveraging the difference in reflectance caused by hemoglobin absorption at 590 nm and applying the derived correction to the measurement at 465 nm, ensuring accurate bilirubin quantification.

## 3. Results

Six non-hemolyzed bilirubin plasma samples, tested in repetitions of five, were used to calibrate the reflectance photometer. Specifically, the curve was constructed using the five bilirubin levels in the panel plus an additional lower level (0.56 mg/dL). An exponential law was used to approximate the relationship between normalized reflectance and bilirubin concentration, yielding a significant correlation (R^2^ > 0.99) (Figure 2).

A total of 125 single tests were performed. The calibration curve was used to calculate bilirubin values for both reference and hemolyzed plasma samples. A significant influence of hemoglobin on the bilirubin results was found, leading to major overestimation errors. Table 2 shows the averaged results without any compensation for each bilirubin level and the associated root mean square error.

**Figure 3:**
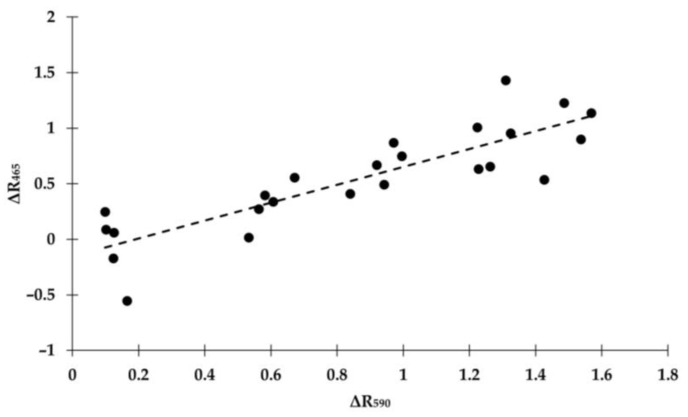
Black dots represent ΔR_465_ and ΔR_590_ for every sample in the test panel. Each point is the mean of the five single tests in the sample. The dashed line represents the linear equation used to approximate the relationship between ΔR_465_ and ΔR_590_.

A significative correlation was found between ΔR_465_ and ΔR_590_, and a linear model adequately approximated the relationship with acceptable accuracy (R^2^ = 0.74, *p* < 0.05) (Figure 3).

**Figure 4:**
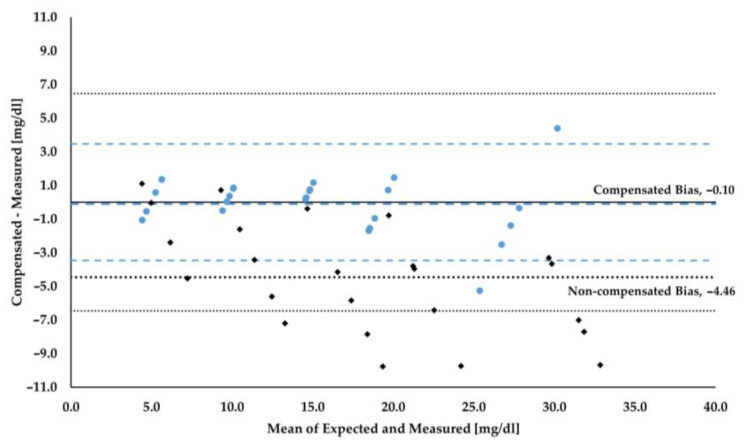
Bland–Altman plot showing both non-compensated and compensated results. Black diamonds represent non-compensated results; blue circles represent compensated results; thin dotted black lines represent upper and lower limits of agreement for non-compensated results (6.46 and −6.46 mg/dL, respectively); thin dashed blue lines represent upper and lower limits of agreement for compensated results (3.46 and −3.46 mg/dL, respectively); thick dotted black line represents bias for non-compensated data (−4.46 mg/dL); thick dashed blue line represents bias for compensated data (−0.10 mg/dL).

The compensation algorithm was applied and the corrected reflectance at 465 nm (R_465, ref_) was calculated using the linear relationship shown in Figure 3. Table 3 presents the mean values and standard deviations for the compensated results, as well as the root mean square errors for comparison with non-compensated data. Figure 4 shows the Bland–Altman plot for both the non-compensated and compensated results. Overall, compensated results show reduced standard deviation values at all bilirubin levels except for the highest. More importantly, however, the root mean square errors are less than half of those without any compensation across all levels tested.

## 4. Discussion

In this study, we assessed the performance of an algorithm designed to compensate for hemoglobin interference in bilirubin measurements using a simple and portable two-wavelength reflectance photometer. This device was capable of performing measurements on 30 µL samples without the addition of reagents. A panel of plasma samples, comprising five different concentrations of bilirubin and five different degrees of hemolysis, was tested, totaling 125 tests. A commercial discrete LED emitting light at 465 nm was used to measure bilirubin concentration, while another discrete LED emitting light at 590 nm was used to estimate hemoglobin concentration, as bilirubin shows no absorption at this wavelength. The proposed algorithm combined reflectance data from the two wavelengths to subtract the influence of hemoglobin on the results.

Non-compensated results showed significant overestimation errors, particularly at higher bilirubin levels, which can lead to the misdiagnosis and potential overtreatment of patients. Compensated results, on the other hand, showed a substantial increase in accuracy at all levels. The highest level, with an expected bilirubin concentration of 28.00 mg/dL, retained a non-negligible RMSE, although it was less than half compared to the non-compensated data (3.33 vs. 6.73 mg/dL). A potential factor to consider is the calibration curve presented in Figure 2. While the exponential model effectively captured the relationship between normalized reflectance and bilirubin, it showed a slight tendency to underestimate the concentration of 4.98 mg/dL and slightly overestimate the concentration of 28.00 mg/dL. However, due to the minimal nature of such deviations, it can be speculated that this aspect did not significantly detract from the overall accuracy of the model and thus of the system. On the whole, the proposed algorithm demonstrated good performance in compensating for hemoglobin interference across a wide range of bilirubin concentrations, covering nearly the entire range of clinical diagnostic interest.

To the best of our knowledge, few recent studies have specifically addressed hemoglobin interference on bilirubin measurements using simple devices, despite its relevance for developing countries and resource-constrained settings. Kudavelly and Balakrishnan proposed a simple, low-cost, and two-wavelength spectrophotometer working with just 5 µL plasma samples and reported hemoglobin compensation capabilities; however, the performance of the device in this regard was not disclosed by the authors [30]. Keahey et al. reported a three-wavelength instrument tailored for low-resource environments like Africa and South Asia, working with 50 µL whole blood samples and featuring one dedicated wavelength for hemoglobin measurement. While positive results in bilirubin determination were demonstrated in a pilot study in Malawi, the specific impact of hemoglobin was not thoroughly discussed [51]. Lastly, Tan et al. proposed a low-cost paper-based assay system with great tolerance to hemoglobin interference and satisfactory accuracy in bilirubin measurement. However, this method was based on Jendrassik–Grof diazotization, requiring the use of reagents before analysis and an external device for quantitative reading, such as a scanner or a smartphone with a camera [52]. These devices show promising potential in terms of performance, low cost, and ease of use—critical features for harsh and under-resourced settings. However, only the latter study disclosed the performance of hemoglobin compensation, though it involved the use of reagents and additional machinery.

We believe that this system, comprising the reflectance photometer and compensation algorithm, could be highly valuable in under-resourced settings, such as LMICs, where the burden of NH is greatest. The system’s evaluation against the WHO’s A.S.S.U.R.E.D. guidelines yield positive outcomes—affordability, the primary criterion, is achieved due to the system’s simplicity and the lack of complex optical or electronic components, as well as the elimination of the need for reagents or sample preparation in real-world applications. The algorithm’s compensation performance ensures that the system meets the required sensitivity and specificity, the second and third criteria, respectively, at least in terms of bilirubin concentration measurement and hemoglobin interference rejection. Additionally, the system satisfies the user-friendly and equipment-free criteria due to its simplicity. The automated nature of the system, which performs analyses and retrieves results in a very short time, further reinforces these advantages.

A limitation of this study was the limited precision of the 590 nm wavelength in estimating hemoglobin concentration, which led to a dispersion of data points, as shown in Figure 2. This limitation in precision did not emerge in our preliminary study, which also investigated the performance of three other wavelengths (570, 575, and 605 nm) [50]. In that work, 590 nm was concluded to be the best overall choice among those tested. This dispersion resulted in higher, although acceptable, standard deviations and root mean square errors for the compensated data.

Future efforts will focus on increasing the precision of hemoglobin estimation and applying the algorithm to higher concentrations of hemoglobin. Subsequently, the reflectance photometer used in this experiment will be evaluated in real-world clinical settings to assess the robustness and usability of this method.

## 5. Conclusions

In this work, an algorithm for hemoglobin compensation was applied to a basic two-wavelength reflectance photometer designed to measure total serum bilirubin concentration in whole blood samples without the use of any reagents. A panel of bilirubin concentrations with various degrees of hemolysis was tested, covering a substantial portion of the clinically significant range for NH. The algorithm effectively reduced overestimation errors by more than half. This study lays the groundwork for subsequent real-world clinical investigations with neonatal blood samples.

## Figures and Tables

**Table 1:** The panel of samples generated for this experiment. For each of the five bilirubin levels, five different levels of hemolysis are produced, and each sample is repeated five times.

Sample #	Bilirubin Concentration [mg/dL]	Hemoglobin Concentration [g/dL]
B1H1–B1H5	4.96	0.06, 0.29, 0.50, 0.75, 0.99
B2H1–B2H5	9.67	0.06, 0.29, 0.50, 0.75, 0.99
B3H1–B3H5	14.47	0.06, 0.29, 0.50, 0.75, 0.99
B4H1–B4H5	19.33	0.06, 0.29, 0.50, 0.75, 0.99
B5H1–B5H5	28.00	0.06, 0.29, 0.50, 0.75, 0.99

**Table 2:** Non-compensated bilirubin results presented as the mean value and standard deviation for each level, as well as root mean square errors with respect to the expected value.

Bilirubin Concentration [mg/dL]	Non-Compensated Bilirubin Measurement [mg/dL]	RMSE [mg/dL]
4.96	7.52 ± 2.92	3.12
9.67	13.09 ± 2.81	2.63
14.47	20.07 ± 3.22	4.43
19.33	24.27 ± 2.99	5.77
28.00	34.27 ± 2.44	6.73

**Table 3:** Hemoglobin-compensated bilirubin results presented as mean values, standard deviations, and root mean square errors for each level with respect to the expected value.

Bilirubin Concentration [mg/dL]	Compensated Bilirubin Measurement [mg/dL]	RMSE [mg/dL]
4.96	5.04 ± 0.95	0.95
9.67	9.98 ± 0.50	0.59
14.47	15.08 ± 0.35	0.70
19.33	18.92 ± 1.26	1.33
28.00	26.97 ± 3.17	3.33

## Data Availability

Relevant data are available upon reasonable request to the corresponding author.
